# Gamification in education—teachers’ perspectives through the lens of the theory of planned behavior

**DOI:** 10.3389/fpsyg.2025.1571463

**Published:** 2025-03-27

**Authors:** Laura Leiss, Jörg Großschedl, Matthias Wilde, Silvia Fränkel, Sebastian Becker-Genschow, Nadine Großmann

**Affiliations:** ^1^Faculty of Mathematics and Natural Sciences, Institute for Biology Education, University of Cologne, Cologne, Germany; ^2^Faculty of Biology, Department for Didactics of Biology, Bielefeld University, Bielefeld, Germany; ^3^Faculty of Mathematics and Natural Sciences, Digital Education Research, University of Cologne, Cologne, Germany

**Keywords:** gamification, theory of planned behavior, intentions, teacher behavior, attitude

## Abstract

**Introduction:**

Gamification has become an important topic in research, as it is increasingly applied in school lessons. However, gamification is still evolving as a research field, and the investigation of the conditions for and effects of its application on students is just beginning. Previous research on teacher-related conditions for the successful implementation of gamification often lacks a theoretical foundation. The current study aims to close this gap by using the theory of planned behavior to explore teacher variables that impact the use of gamification in class.

**Methods:**

For this purpose, 196 teachers (41.80 ± 10.90 years; 70.9% female) were surveyed regarding the constructs anchored in this theory in an online questionnaire.

**Results and discussion:**

The results reveal that teachers’ attitude toward gamification, their perceived subjective norm, and their perceived self-efficacy regarding the implementation of gamification are important predictors of their intention to use gamification in class. However, none of these variables predicted the actual application of gamification in class. Instead, we found mediating effects of teachers’ intention in the relationship between their attitude and behavior. In addition, moderating effects of self-efficacy were observed in the relationship between attitude and intention. Beyond these moderations, intention was found to be a direct predictor of behavior. Our results provide important insights for promoting the application of gamification in schools and for designing teacher training measures.

## 1 Introduction

Interest in gamified learning methods, known as gamification, game-based learning, or serious games, has been growing since the beginning of the 21st century (Bourgonjon et al., 2013; Kalogiannakis et al., 2021). These learning methods are applied in class to enable motivating and cooperative learning with (digital) game elements (Fotaris and Mastoras, 2020; Ouariachi and Wim, 2019). It is assumed that these learning methods give students the opportunity to control their own learning process and positively affect their performance (Fischer and Reichmuth, 2020; O’Brien and Pitera, 2019). The positive effects on learning are mainly attributed to *immersion*, a state of intense mental involvement (Vaz de Carvalho and Coelho, 2022). Most importantly, gamified learning methods are designed to strengthen 21st century skills, such as problem solving, critical thinking, collaboration, and creativity (O’Brien and Pitera, 2019; Sterel et al., 2018). These skills prepare students for successfully mastering challenges in school and beyond (O’Brien and Pitera, 2019). A further advantage of gamified learning methods is that they can be implemented in both analog and digital form, thus meeting the demand to incorporate new media in class (Drossel et al., 2020; Serdyukov, 2017) and to address the “digital generation” (Hilton and Honey, 2011). This often presents teachers with major challenges, such as the required time and limited resources (Aguaded-Gómez, 2011; Fotaris and Mastoras, 2020; Siregar et al., 2023).

Given the potential of gamified learning methods for important educational outcomes and the needs of students in an increasingly digital world, teachers’ interest in using these concepts in their lessons is on the rise (Baek et al., 2015), despite Fischer and Reichmuth (2020) noting that teachers are often not motivated to apply such concepts in their lessons. However, teachers’ interest and motivation are not the only variables that are important in making the benefits of gamified learning methods accessible to students. For example, the decision to use such innovative concepts depends on the acceptance of the environment in which a teacher works (Bourgonjon et al., 2013; Parra-González et al., 2021).

Sajinčič et al. (2022) emphasizes the importance of considering such theoretical frameworks for a more systematic approach to studying conditions for the use of gamification in class. Adopting this theory-based perspective is the aim of the present study. One theory that provides a comprehensive insight into significant variables related to intentions and behavior is the theory of planned behavior (TPB) (Ajzen, 2020). By incorporating this theory, the current study investigates the conditions for the implementation of gamification in class. According to TPB, teachers’ attitude toward gamification, their perception of the subjective norm of gamification as well as their perception of behavioral control over implementing gamification essentially influence intentions and behavior (see Ajzen, 2020). In the investigation of these variables and assumptions, both theoretically and empirically based mediations and moderations are considered.

## 2 Theory

### 2.1 Defining gamification

Gamification, game-based learning, or serious games refer to gamified concepts that can be used in various contexts. In addition to the use of gamified processes in companies, gamified learning methods are increasingly being used in education, so far mainly in the field of higher education (Fischer and Reichmuth, 2020; Stieglitz, 2017). It is assumed that the use of these concepts can promote motivation and performance as well as develop skills necessary for future challenges (Fotaris and Mastoras, 2020).

There is no uniformly recognized definition of gamification in the literature (Cheng, 2020; Sailer et al., 2016). According to Deterding et al. (2011), it is the application of game design elements in contexts that are atypical for gaming. Taking Werbach’s (2014) perspective into account, gamification can be described as the process of designing game-like activities in a non-game context. The definition applies to both analog and digital activities (see Fischer and Reichmuth, 2020; Sailer et al., 2016). In the design of these activities, elements are used that are present in most entertainment games (Deterding et al., 2011). Frequently mentioned elements are leaderboards, badges, social engagement, feedback, points, and storyline (Dicheva et al., 2015).

In addition to the use of game design elements, (entire) games represent game-based learning, which are usually integrated into lessons in digital form Jacob and Teuteberg (2017). Due to the lack of differentiation between game-based learning and serious games, the latter term is sometimes used synonymously with game-based learning. Both terms include the use of an entire game as opposed to the use of gamified elements in a gamification approach (Fischer and Reichmuth, 2020). Although game-based learning and serious games are related concepts, they differ in their basic approach and application. In game-based learning, games originally developed for entertainment purposes, such as strategy games or action games, are used to promote long-term thinking or to teach motor skills and reaction times (Jacob and Teuteberg, 2017). According to Jacob and Teuteberg (2017), the game content of serious games is closely linked to the learning objectives. In serious games, reality is experienced in a computer-supported, game-like way, for example, through virtual operating rooms (Jacob and Teuteberg, 2017).

The current study refers to the implementation of gamified elements and mechanisms in non-game-based contexts. Therefore, the term *gamification* is retained throughout the article. As our study focuses on primary and secondary education, the following section will discuss gamification in this context.

### 2.2 Applying gamification in class

The use of gamification offers a variety of benefits, including the promotion of 21st-century skills, which are becoming increasingly important in a rapidly changing world. These include critical thinking, collaboration, creativity, and problem-solving skills (Battelle for Kids, 2019; Sterel et al., 2018). In addition to fostering 21st-century skills, the integration of gamification can increase motivation to learn, support collaborative work, and lead to better learning outcomes (Al-Azawi et al., 2016; Kim and Castell, 2021; Parra-González et al., 2021; Yildrim, 2017). Nevertheless, gamification cannot be viewed uncritically, as there is empirical evidence of negative effects, albeit limited (Almeida et al., 2023; Rapp et al., 2019; Toda et al., 2018). Almeida et al. (2023) identified research that revealed negative effects of game design elements, including leaderboards or competitions, which can lead to dislike, demotivation, and loss of performance. Despite these suspected negative effects, certain forms of gamification have been shown to be effective in increasing motivation and engagement (Ouariachi and Wim, 2019). One type of gamification that is increasingly attracting attention in education are escape games, in which participants have to solve riddles and puzzles in order to escape a game environment within a set time frame (Fotaris and Mastoras, 2020; Grande-de-Prado et al., 2021; Veldkamp et al., 2020).

There are many approaches to the empirical evaluation of the effects of gamification on student variables (Ozcinar et al., 2019; Queiruga-Dios et al., 2020; Van Roy and Zaman, 2018). Although teachers play a key role in the use of gamification, only a few studies take the teachers’ perspective in gamification research (Martí-Parreño et al., 2016). However, for students to benefit from the positive effects of gamification (Bourgonjon et al., 2013; O’Brien and Pitera, 2019), such concepts must be applied by their teacher in class. Sajinčič et al. (2022) point out that the actual use of gamification by teachers in the classroom is rare. Concerns about negative effects on their students’ learning process (Almeida et al., 2023) could be one of the reasons why teachers do not use gamification concepts in their lessons (Jääskä and Aaltonen, 2022; Pozo et al., 2022). To test such assumptions and gain a comprehensive, theoretically sound, and empirical insight into the formation of intentions and the behavior of teachers regarding gamification in the classroom, we designed the current study. As we used the TPB as theoretical framework for our study, the variables anchored in this theory are discussed in more detail in the following sections.

### 2.3 Theory of planned behavior (TPB)

**Figure 1:**
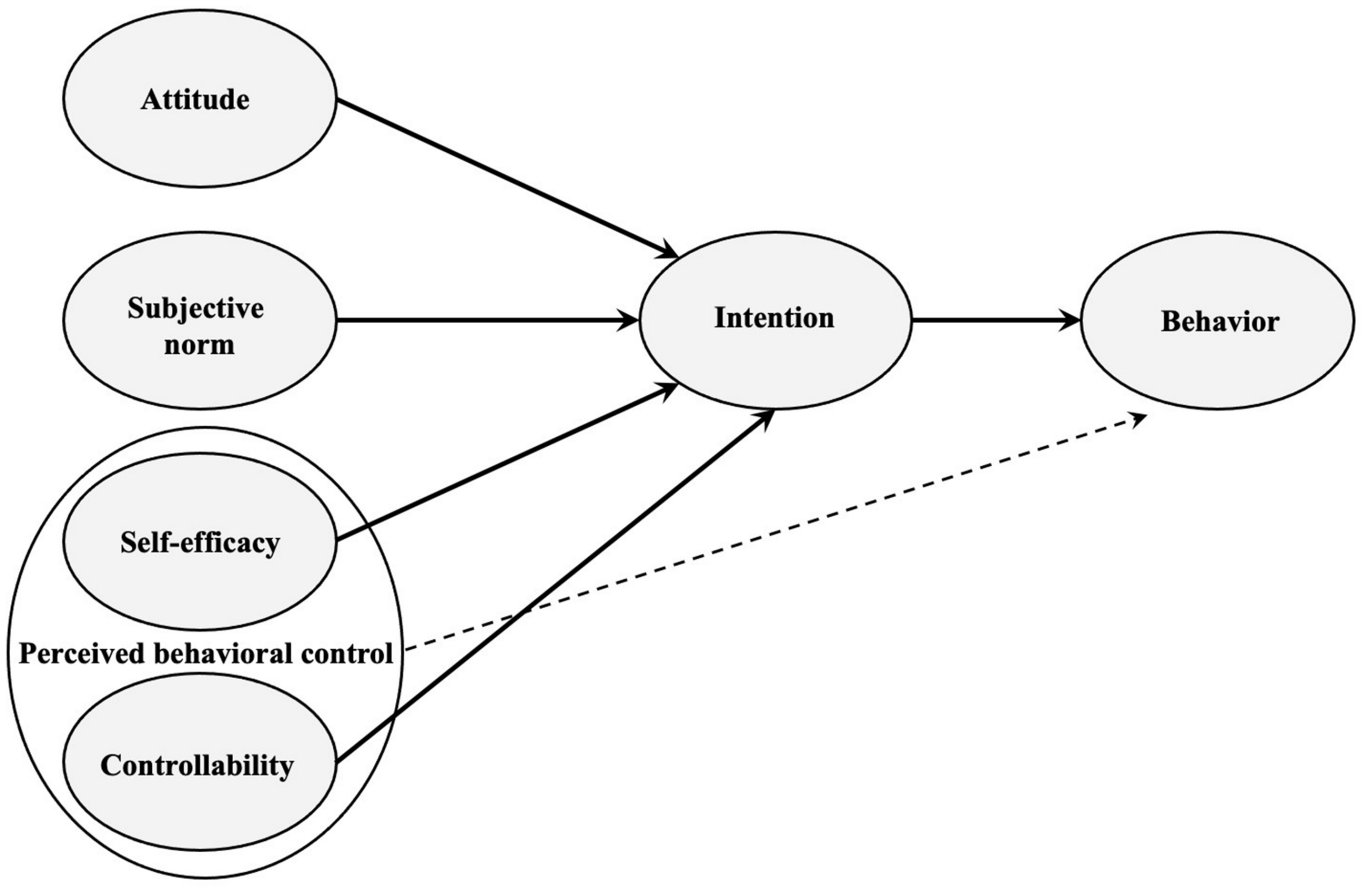
Assumed relationships in the theory of planned behavior (adapted based on Ajzen, 1991, 2020).

TPB proposes a model that can be used to predict and explain human behavior in specific contexts (Ajzen, 1991). According to Karaböcek and Erb (2013), it is the best operationalized and most empirically tested theory in social psychology, which is also applied in educational research as a basis for research into the behavior of teachers. In a meta-analysis of 185 independent studies, Armitage and Conner (2001) were able to show that 39% of the variance in the intention to perform a certain behavior is explained by the variables covered by the TPB. These variables include the attitude toward the respective behavior, the perceived subjective norm regarding the behavior, and the perceived behavioral control (Ajzen, 1991; Graf, 2007; see also Figure 1). These variables cannot be considered independently of each other (Ajzen, 1991; Graf, 2007).

The distinction between the attitude toward the behavior and the subjective norm regarding the behavior is a central feature of the TPB and stems from the theory of reasoned action (Ajzen and Fishbein, 1980; Ajzen, 1991; Rossmann, 2021). In contrast to the theory of reasoned action, the TPB considers perceived behavioral control, since the prediction of attitude and subjective norm can only be accurately estimated if the behavior is completely under voluntary control (Graf, 2007). The three variables determine behavioral intention as an immediate precursor of the behavior in question (Ajzen, 2020). The stronger the intention to perform the respective behavior, the higher the probability that the behavior will follow (Ajzen, 2020).

In the following sections, these three variables are examined in more detail, considering current findings in the school context. As there are only few empirical findings for some of the variables in the context of gamification in school, it was sometimes necessary to adopt a broader perspective.

#### 2.3.1 Attitude toward behavior

Attitude toward a behavior refers to how positively or negatively a person evaluates the behavior in question (Ajzen, 1991). Attitudes toward a particular behavior arise from an individual’s beliefs about the likely consequences of that behavior; these are referred to as behavioral beliefs (Ajzen and Kruglanski, 2019). They act as multipliers in forming an attitude toward the behavior (Ajzen and Kruglanski, 2019; Ajzen, 2020). Thus, attitude results from the total number of subjectively evaluated behavioral beliefs (Ajzen, 2005; Graf, 2007).

In the context of gamification, different attitudes of teachers have been identified to date. While some research has found a preference among educators for implementing gamification in the classroom, other studies have found contrary results (Becker, 2007; Martí-Parreño et al., 2016; Kepceoğlu, 2019; Sajinčič et al., 2022). Sajinčič et al. (2022) showed that teachers’ attitudes toward gamification are strongly positive (see also Martí-Parreño et al., 2016). However, it should be noted that experienced teachers tend to have a more positive attitude than inexperienced teachers (Sajinčič et al., 2022). According to Becker (2007), gamified approaches are often perceived as annoying. Some concerns and limitations regarding gamification that Kepceoğlu (2019) found in their survey of prospective teachers are the loss of student motivation due to repeated incorrect answers, possible technical problems with the application, and a high expenditure of time with little increase in knowledge.

A strong relationship between attitude and behavioral intention has been shown not only in the context of gamification, but in other areas as well (Wallace et al., 2005; Sánchez-Mena et al., 2016; Sánchez-Mena et al., 2017; Sajinčič et al., 2022). Looking at behavior rather than intention, Martí-Parreño et al. (2016) found a discrepancy between university lecturers’ attitude and their actual use of gamification. Despite the lecturers’ positive attitudes toward gamification, only a small percentage regularly used gamified approaches in their courses (Martí-Parreño et al., 2016). Studies based on TPB from other contexts suggest that a positive attitude toward the behavior in question does not necessarily lead to the behavior itself. For example, this *attitude-behavior gap* has been observed in the context of organ donation (Basten, 2013) as well as inclusive education and the teaching of appropriate behaviors (Gülsün et al., 2023). Besides attitude, further variables, such as the subjective norm, play a significant role in shaping behavior, as discussed in the following section.

#### 2.3.2 Subjective norm regarding behavior

The subjective norm regarding behavior refers to the social pressure exerted on an individual to engage in or refrain from a specific behavior (Ajzen, 1991). Fishbein and Ajzen (2010) distinguish between two sources of this social pressure: injunctive and descriptive normative beliefs. Injunctive normative beliefs are formed based on expectations that a reference person or group approves or disapproves of the behavior (Ajzen, 2020). Descriptive normative beliefs, on the other hand, are formed when attachment figures or reference groups perform the behavior themselves (Fishbein and Ajzen, 2010). Together, these two types of normative beliefs contribute to the overall perceived social pressure to act according to the subjective norm (Ajzen, 2005; Graf, 2007; Ajzen, 2020).

In general, studies identify the subjective norm as an important variable influencing teachers’ acceptance of behaviors in their teaching practice (Hsu and Lu, 2004; Teo et al., 2016). However, inconsistencies exist regarding the predictive effect of the subjective norm on behavioral intention. Some studies indicate a relationship between the subjective norm and the intention to use gamified approaches in the classroom (Bourgonjon et al., 2013; see also Fischer et al., 2018; Rossmann, 2021). In the context of sustainability, Weber and Fiebelkorn (2019) demonstrated a significant positive effect of the subjective norm on the intention of pre-service biology teachers to eat sustainably. Nevertheless, subjective norm is considered the weakest predictor compared to the variables of attitude and perceived behavioral control (Weber and Fiebelkorn, 2019). In the study conducted by Lübke et al. (2016), the subjective norm was not confirmed as a predictor of the behavioral intention to teach inclusively. It is important to note that Lübke et al. (2016) focused on the head teacher as the meaningful person rather than colleagues. Teo et al. (2016) found a negative impact of the subjective norm on behavioral intention. In their study, the perception and expectations of other people (e.g., colleagues and parents) negatively influenced teachers’ intention to use technology in the classroom (Teo et al., 2016). Moreover, the effect of subjective norm on behavioral intention across different class levels was inconsistent (Teo et al., 2016; see also Hou et al., 2022). Studies that simultaneously examine both intention and behavior are rarely found in the educational context.

The effects of teachers’ perceived subjective norm on their intention—and, according to the TPB, on their behavior—can also be assumed for gamification. Teachers are influenced by their social environment and the expectations of others (Bourgonjon et al., 2013). This suggests that their opinion about gamification is not independent of their social environment or cultural influences (AlMarshedi et al., 2017; Asiri, 2019). However, when teachers are asked about their perception of gamification as the norm, only moderate agreement is observed (Bourgonjon et al., 2013).

#### 2.3.3 Perceived behavioral control

Perceived behavioral control describes the extent to which an individual feels capable of controlling the performance of a behavior (Ajzen, 1991). Both the perception of personal and environmental factors that may facilitate or hinder behavioral attempts contribute to the perception of behavioral control (Ajzen, 1991; Ajzen, 2020). These factors, which primarily stem from previous experiences or anticipated obstacles, influence whether an individual evaluates the behavior as easy or difficult (Ajzen, 1991; Fishbein and Ajzen, 2010). Personal factors include individuals’ perception of their ability to perform the behavior (Ajzen, 1991), often referred to as control beliefs (Ajzen, 2020). Control beliefs directly affect perceived self-efficacy and, consequently, an individual’s sense of control over performing a behavior (Fishbein and Ajzen, 2010). Additionally, individuals’ subjective assessment of environmental factors—such as conditions that facilitate or hinder the behavior— shapes their perceived behavioral control (Ajzen, 2002). Thus, perceived behavioral control results from an interplay of various factors (Ajzen, 2005; Ajzen, 2020; Graf, 2007). In terms of assessing this variable, it is often operationalized through two dimensions: controllability, which addresses environmental conditions, and self-efficacy, which focuses on personal conditions (Bandura and Adams, 1977; e.g., Francis et al., 2004).

According to Ajzen (1991), perceived behavioral control can exert both direct and indirect influences on behavior. However, a direct prediction of the behavior in question may be inaccurate if a person has little information about the behavior, new situational demands arise, or the resources for performing the behavior have changed (Kan and Fabrigar, 2017). Therefore, the relationship between perceived behavioral control and behavior is depicted with a dashed arrow in Figure 1.

Research on teachers generally indicates that perceived behavioral control is a significant predictor of behavioral intention (Gülsün et al., 2023; Klassen and Tze, 2014); however, these studies do not specifically address intentions to apply gamification. In a systematic meta-analysis, Klassen and Tze (2014) found a strong relationship between self-efficacy and the behavior of both prospective and experienced teachers in the classroom. They identified that higher perceived self-efficacy is associated with more positive teacher behaviors, including teaching effectiveness and student evaluations. Similarly, Gülsün et al. (2023) demonstrated that teachers’ behavior, especially the ability to convey appropriate behaviors to students, is influenced by their self-efficacy in dealing with students’ behavior management. Consistent with these findings, Teo et al. (2016) found that perceived behavioral control plays a crucial role in explaining teachers’ intention to use technology in the classroom. Wang and Tsai (2022) examined teachers’ behavioral intention to engage in school disaster preparedness, as well as their actual behavior, and found perceived behavioral control to be a predictor of both variables.

Besides direct effects on intentions and behavior, there is growing evidence of moderating effects of perceived behavioral control (Ajzen, 2020; Hagger et al., 2022). In Hagger et al.’s (2022) more recent study, perceived behavioral control did not influence the effects of attitude and subjective norm on behavioral intention but moderated the relationship between behavioral intention and behavior. This connection between behavioral intention and behavior will be explored in more detail in the next section.

#### 2.3.4 Behavioral intention

Behavioral intention is composed of motivational factors that influence behavior and determine the likelihood of performing the behavior in question, provided it is under volitional control (Ajzen, 1991; Graf, 2007). Ajzen (1991) posits the assumption that “the stronger the intention to engage in a behavior, the more likely should be its performance” (p. 181). Thus, an individual’s actual behavior is predicted by his/her behavioral intention, that is, his/her personal motivation (Ajzen, 1991). According to the TPB, behavioral intentions are shaped by the three aforementioned variables attitude toward the behavior, subjective norm regarding the behavior, and perceived behavioral control (Ajzen, 2020).

Some studies in the educational context have investigated behavioral intentions rather than actual behavior and the relationship between the two variables (Lübke et al., 2016; Weber and Fiebelkorn, 2019; Urton et al., 2023). However, a key feature of the TPB is the direct link between behavioral intention and behavior (Ajzen, 1991; Fischer et al., 2018). Knauder and Koschmieder (2019) point to contradictory results regarding the relationship between behavioral intention and actual behavior in educational contexts. According to Fischer et al. (2018), behavioral intention predicts behavior in most independent studies with medium to large effect sizes. Reference is made here to the meta-analyses by Armitage and Conner (2001) and Rendall and Wolff (1994) (*r* = 0.47 and *r* = 0.45). In contrast, Sheeran (2002) found in his analysis of meta-analyses that people are often unable to translate behavioral intentions into actual behavior (see also Gollwitzer et al., 2009). Additional strategies are required to overcome this discrepancy, the intention-behavior gap (Sheeran, 2002).

Previous studies found relationships between intentions and behavior in the educational context. Wang and Tsai (2022), for example, were able to show a significant direct effect of the behavioral intention of teachers to participate in school disaster preparedness on the actual behavior to become more involved in preparing for a school disaster. Chu and Chen (2016) assumed that a higher behavioral intention with regard to e-learning is related to more frequent use. However, in their study, they only found a weak relationship between intention and actual use of e-learning technologies. As most studies focus on behavioral intention, we are not aware of any studies on the lack of connection between behavioral intention and behavior in the school context (see Hassan et al., 2016; Armitage and Conner, 2001).

In addition to the direct link between intentions and behavior, behavioral intention as a direct precursor of behavior can mediate the impact of attitude, subjective norm, and perceived behavioral control on behavior (Ajzen, 2020; Chen, 2018). However, the mediating effect of behavioral intention on the relation between these three variables and behavior has not yet been thoroughly investigated in the context of gamification in education (see Chen, 2018). This research gap was considered in our study as well.

## 3 Research goal

Previous research in the context of gamification has most often neglected the role of the teacher or has considered teacher variables with insufficient theoretical foundation. In the current study, we aimed to gain a theoretically sound and comprehensive insight into teacher variables that have an impact on the application of gamification in class. Based on TPB (Ajzen, 2020), we assumed that attitudes, perceived subjective norm, and perceived behavioral control positively influence the intention to use gamification and, consequently, the actual use of gamification. Moreover, previous research suggests mediating effects of intention and moderating effects of perceived behavioral control in these relationships (Ajzen, 2020; Chen, 2018; Hagger et al., 2022).

**Figure 2:**
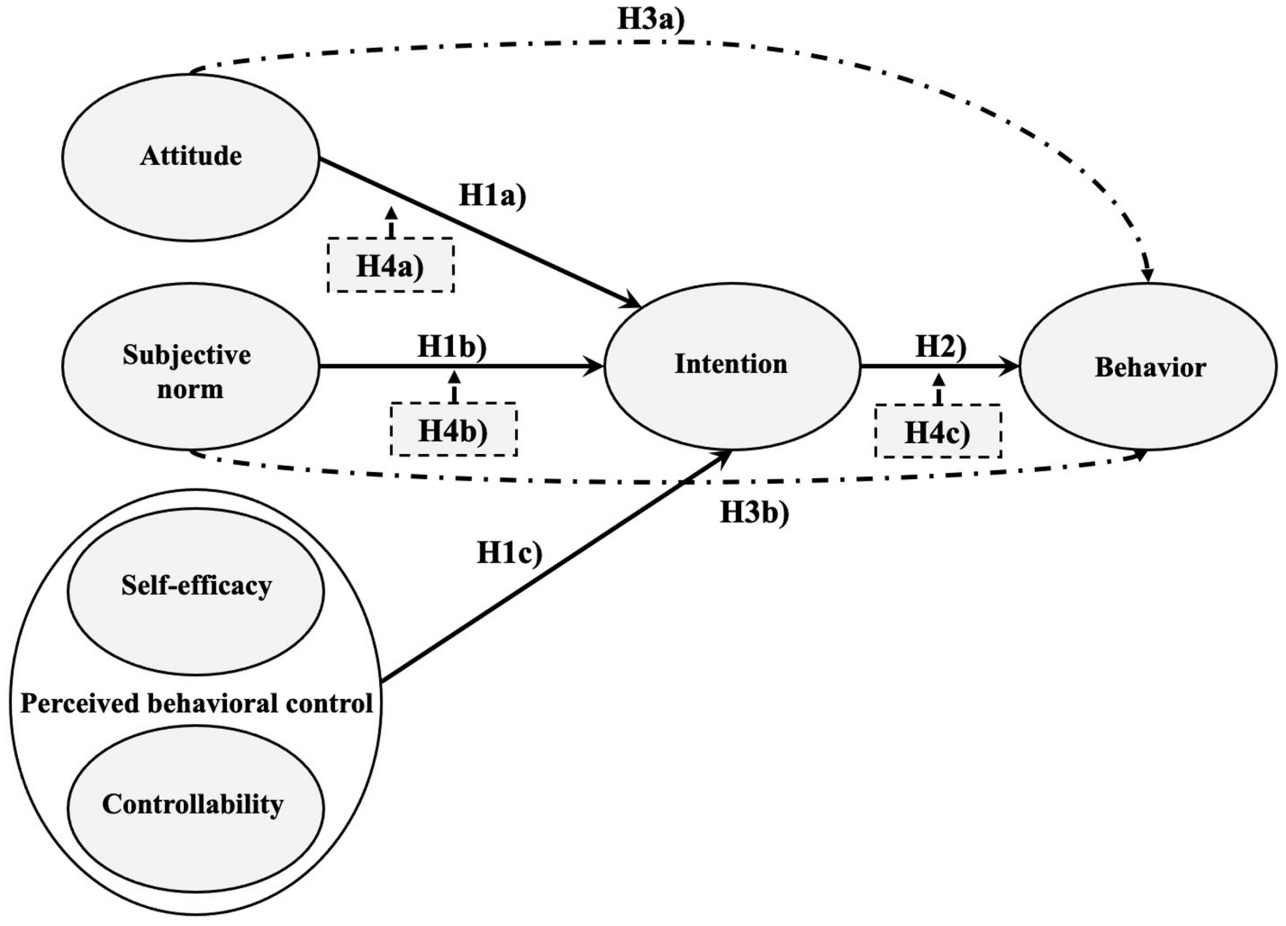
Theoretical model supplemented with the hypotheses of the current study. Mediating effects are shown by the dashed and dotted lines whereas moderating effects are indicated by the boxes with arrows.

To address these relationships, we tested the following hypotheses (Figure 2):

*H1*: The intention to use gamification in the classroom is predicted by:

*H1a*: The attitude toward the use of gamification in the classroom.

*H1b*: The subjective norm of using gamification in the classroom.

*H1c*: The perceived behavioral control regarding the use of gamification in the classroom.

*H2*: Behavior is predicted by the intention to use gamification in the classroom.

*H3*: Behavior is indirectly (via intention) predicted by:

*H3a*: The attitude toward the use of gamification in the classroom.

*H3b*: The subjective norm of using gamification in the classroom.

*H4*: Perceived behavioral control regarding the use of gamification in the classroom is a moderator of the relationship:

*H4a*: Between the attitude toward the use of gamification in the classroom and the intention to use gamification.

*H4b*: Between the subjective norm of using gamification in the classroom and the intention to use gamification.

*H4c*: Between the intention to use gamification in the classroom and behavior.

## 4 Materials and methods

### 4.1 Sample

In the current study, 207 participants voluntarily filled out an online survey, of which 196 were ultimately included in the analysis. The reasons for the exclusion of 11 people are outlined over the course of this chapter. Before participation, all participants provided informed consent. Completing the online questionnaire took approximately 15 min.

The average age of the 196 investigated participants was 41.80 years (*SD* = 10.90 years), ranging from 24 years (minimum) to 64 years (maximum). Seventy point nine % of these participants classified themselves as female, 28.6% as male, and 0.5% chose not to specify their gender. Participants reported an average of 13.19 years of professional experience (*SD* = 9.25 years), with a range of zero years (minimum) to 38 years (maximum). One participant provided an implausible value for their professional experience and was excluded from the analyses related to this variable.

Seventy-six point five % of the teachers surveyed teach a STEM subject, 44.4% teach a language, 25.5% teach a social science subject, and 15.3% teach physical education (multiple answers were possible). One of the participants did not specify their teaching subjects. Most participants (72.4%) teach at higher track secondary schools (“Gymnasium” or “Gesamtschule”), 14.7% teach at lower track secondary schools (“Realschule,” “Hauptschule,” or “Sekundarschule”), 4.1% teach at a special needs school, 4.6% at an elementary school, and 4.1% at a vocational college.

### 4.2 Questionnaire

Before completing the questionnaire, the teachers were given a brief description of the term “gamification” to ensure that all participants shared a common understanding. The definition was as follows: “Gamification means that playful elements are transferred to non-game contexts such as the classroom. Escape games, quiz games or game-based simulations are examples of “gamification”. To investigate the variables anchored in the TPB, items were developed using the manual *Constructing Questionnaires Based on the Theory of Planned Behavior - A Manual for Health Service Researchers* by Francis et al. (2004) and the classroom-adapted version by Aptyka and Großschedl (2022). A five-point rating scale was employed to assess these items (1 = totally disagree to 5 = totally agree; example for *attitude*: 1 = useless to 5 = useful).

Attitude was measured using five items, while subjective norm was assessed through three items (Aptyka and Großschedl, 2022; Francis et al., 2004). Perceived behavioral control was investigated indirectly by examining controllability and self-efficacy (Aptyka and Großschedl, 2022; Francis et al., 2004). Controllability was assessed with three items, while self-efficacy was measured using six items (Aptyka and Großschedl, 2022; Francis et al., 2004; Jerusalem et al., 2009). Indications for the validity of the applied scales can be derived from earlier studies (see Aptyka and Großschedl, 2022; Lee et al., 2010; Luszczynska et al., 2005; Rothland, 2013; Scholz et al., 2002) as well as the analyses in our study (see Hartig et al., 2020).

Behavior was surveyed using a self-developed item. Participants were initially asked to indicate, using a three-point rating scale, whether they use gamification in the classroom (“yes,” “no,” or “unsure”). Subsequently, participants were asked to explain their choice. Based on these answers, some of the participants who selected unsure were reassigned to either the “yes” or “no” categories. Participants whose responses could not be clearly classified as either “yes” or “no” were excluded from the analyses presented in this manuscript. A total of seven participants was not included in the analysis because they answered “unsure” (result: 200 instead of 207 participants).

Details of all test instruments, example items, and internal consistencies are depicted in Table 1. Internal consistency was found to range from acceptable to excellent (Hayes and Coutts, 2020).

**Table 1:** Applied scales with number of items, example items, and internal consistency.

Scale	Number of items	Example item	McDonald’s **ω**
Attitude	5	Using gamification in class is (1) useless to (5) useful for students’ learning process.	0.92
Subjective norm	3	My environment expects that I use this method in my teaching.	0.75
PBC – Self-efficacy	6	I am confident that I can use this method in my teaching if I want to.	0.84
PBC – Controllability	3	Whether I use this method in my teaching is entirely my decision.	0.63
Intention	5	I plan to teach my students in this way in the future.	0.93
Behavior	1	I use gamification in my teaching.	

PBC = Perceived Behavioral Control; all items were rated on a 5-point rating scale ranging from 1 to 5.

### 4.3 Data analysis

For our preliminary analyses, we calculated correlations between all investigated variables anchored in the TPB as well as the collected demographic data. Pearson correlations were calculated for correlations between continuous variables, while Spearman correlations were applied for correlations between continuous and categorial variables as well as for correlations between the dichotomous variable behavior and categorial variables (Table 2). Cramer’s V was used for the correlation between two categorial variables.

**Table 2:** Means, standard deviations, and correlations between all latent variables.

	*M*	*SD*	1	2	3	4	5	6	7	8
1. Attitude	4.24	0.68								
2. PBC – Self-efficacy	3.69	0.68	0.26**							
3. PBC – Controllability	4.29	0.78	−0.03	0.06						
4. Subjective norm	2.09	0.84	0.23**	0.30**	−0.28**					
5. Intention	3.59	0.83	0.51**	0.58**	−0.09	0.39**				
6. Behavior	--	--	0.23**	0.26**	0.07	0.13	0.23**			
7. School type	--	--	−0.17*	0.06	−0.24**	0.22**	0.12	0.15		
8. Gender	--	--	−0.16*	−0.16*	−0.03	0.04	−0.14	0.29**	0.37**	
9. Professional experience	--	--	−0.03	−0.10	0.08	−0.11	−0.26**	0.05	0.20	0.13

*N* = 196; *p < 0.05, **p < 0.01; PBC = Perceived Behavioral Control; Behavior: 1 = No, 2 = Yes; School type (1 = “Gymnasium”; 2 = “Gesamtschule”; 3 = “Realschule”; 4 = “Sekundarschule”; 5 = “Hauptschule”; 6 = vocational college; 7 = special needs school; 8 = elementary school); Gender (1 = female; 2 = male; 3 = diverse; 4 = no specified); Professional experience was categorized (1 = < 5 years; 2 = 6–15 years; 3 = > 15 years); continuous variables: rating scale ranging from (1) totally agree to (5) totally disagree.

After a detailed analysis of univariate and multivariate outliers (Aguinis et al., 2013; Leys et al., 2013; Mitchell and Krzanowski, 1985; Tukey, 1977), four multivariate outliers were identified and omitted using Mahalanobis distance analysis (result: 196 instead of 200 participants; chi-square at *p* = 0.001). For the subsequent data analysis, we used R Studio with the package lavaan (Rosseel, 2012). We used a factor score regression method with path analysis and mediation to test the hypotheses (Devlieger and Rosseel, 2017). We applied the approach recommended for a two-stage procedure by first validating the measurement model and then using a mediated path model with factor scores to test our hypotheses (Anderson and Gerbing, 1988). In addition to the assumed mediations, the moderating effects of self-efficacy and controllability—as representatives of perceived behavioral control—were examined (see Ajzen, 2020; Hagger et al., 2022). Due to the categorical nature of some of the data, the unweighted least squares-mean and variance were used as estimator (DiStefano and Morgan, 2014).

Fit indices were used to check how well the model aligned with the empirical data (Hooper et al., 2008; Hu and Bentler, 1999; Kline, 2023; Moosbrugger and Schermelleh-Engel, 2012). The following indices were calculated: the Standardized Root Mean Square Residual (SRMR), the Root Mean Square Error of Approximation (RMSEA), the Comparative Fit Index (CFI), and the Tucker-Lewis Index (TLI). Model fit was evaluated based on established benchmarks. SRMR and RMSEA values below 0.05 indicate a good model fit, while values below 0.10 and 0.08, respectively, are indicative of an acceptable model fit (Hooper et al., 2008; Moosbrugger and Schermelleh-Engel, 2012; Kline, 2023). According to Hu and Bentler (1999), CFI values above 0.95 are considered a good model fit and values above 0.90 indicate an acceptable model fit. Similarly, TLI values above 0.90 reflect a good model fit, whereas values above 0.95 are considered an acceptable model fit (Hooper et al., 2008; Hu and Bentler, 1999).

## 5 Results

### 5.1 Preliminary analyses

As part of the preliminary analyses, we examined the relationships between the investigated variables using correlation analyses (Table 2). We found significant relationships between all investigated variables, except for the relationship between subjective norm and behavior and the relationships with the controllability variable. Controllability was found to correlate significantly only with subjective norm. Further significant correlations were observed among the other determinants of intention and behavior, specifically attitude, self-efficacy, and subjective norm. The strongest significant correlation emerged between self-efficacy and intention. Notably, all reported relationships were positive, except for the relationship between controllability and subjective norm.

In addition to examining correlations among the variables anchored in the TPB, we examined whether these variables correlate with school type, gender as well as professional experience (Table 2). The type of school correlated positively with subjective norm as well as negatively with attitude and controllability. Gender exhibited negative correlations with attitude and self-efficacy as well as positively with behavior. However, in the relationship between gender and behavior, it must be taken into account that Cramers V cannot take on negative values. Last, professional experience was found to negatively correlate with behavioral intention.

### 5.2 Measurement and factor score path model

Before testing our hypotheses, we evaluated the measurement model as a first step. The measurement model showed a central χ^2^ distribution (χ^2^ (215) = 243.23, *p* = 0.091). The values of the absolute fit indices SRMR at 0.049 (< 0.05) and RSMEA at 0.023 (< 0.05) as well as the values of the comparative fit indices CFI at 0.993 (> 0.95) and TLI at 0.992 (> 0.95) indicated a good model fit for the proposed measurement model. Given the satisfactory results of the measurement model, we proceeded to the second step, which involved the factor score path analysis. When analyzing the path model (χ^2^ (8) = 25.51, *p* = 0.001), we found a SRMR value of 0.021 (< 0.05) and a RMSEA value of 0.037 (< 0.05), indicating a good model fit. Regarding the comparative fit indices, a value of 0.983 (> 0.95) was found for the CFI whereas a value of 0.954 (> 0.95) was found for the TLI. In summary, the fit indices of both the measurement model and the factor score path model indicated a satisfactory goodness of fit, confirming the suitability of the proposed model.

**Figure 3:**
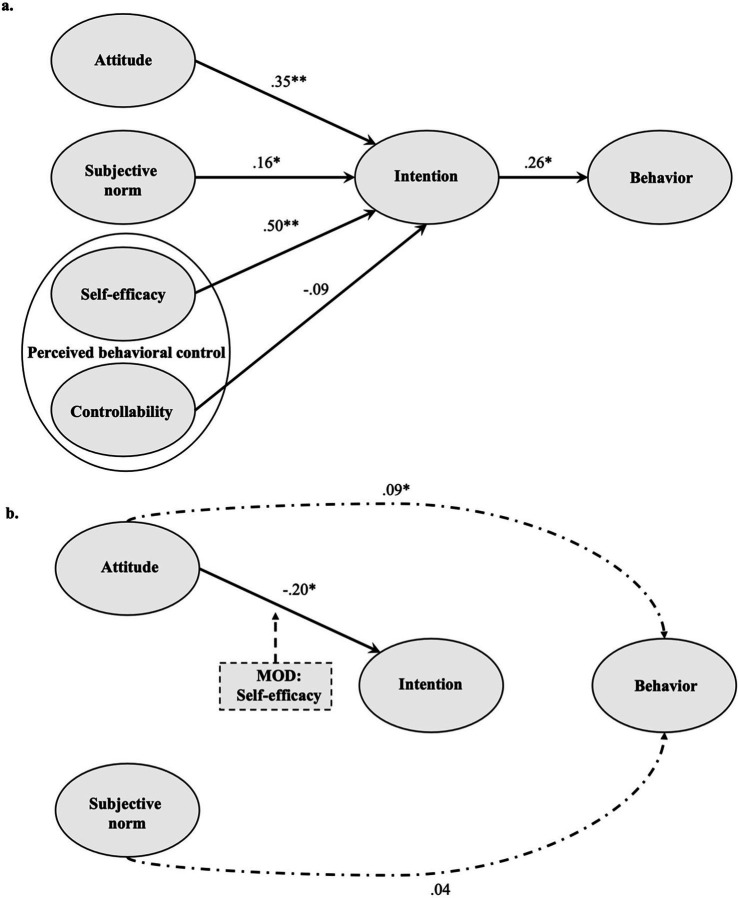
**(a)** Results of the factor score path model. *N* = 196; * *p* < 0.05; **p < 0.001. **(b)** Results of the mediation (dashed and dotted lines) and moderation (MOD; boxes with arrows) analysis. For reasons of clarity, only significant paths are presented.

After confirming the model fit, the individual paths within the model were examined (Figure 3). The findings revealed significant relationships between several variables and the intention to use gamification. Specifically: Attitude had a significant positive effect on intention (β = 0.35, *p* < 0.001, 95%-CI [0.26, 0.46]). Subjective norm also positively influenced intention (β = 0.16, *p* = 0.004, 95%-CI [0.05, 0.26]). Self-efficacy showed the strongest positive effect on intention (β = 0.50, *p* < 0.001, 95%-CI [0.38, 0.62]). In contrast, controllability demonstrated a small but non-significant negative impact on intention (β = −0.09, *p* = 0.051, 95%-CI [−0.17, 0.00]). These variables were predictive of 57.6% of the variance in intention. This corresponds to a high goodness-of-fit in terms of variance explanation (Cohen, 1988). Besides these effects on intention, the intention to use gamification significantly influenced behavior (β = 0.26, *p* = 0.008, 95%-CI [0.03, 0.17]). Intention accounted for a moderate amount of variance (12.3%) in behavior, representing a moderate level of explanatory power.

### 5.3 Mediation and moderation analyses

The analysis focusing on intention as mediator revealed a significant partial mediation effect only for the relationship between attitude and behavior (Table 3). However, no such mediation effects were found for the relationship between subjective norm and behavior, with intention as the mediating variable.

**Table 3:** Indirect effects for the mediation of intention and moderating effects of perceived behavioral control.

Effect type	Relationships	Standardized regression coefficient	Standard error	*p*	*z*	95%-CI [LB, UB]
Mediation-Partial indirect	SN → I → B	β = 0.04	0.01	0.058	1.89	[−0.00, 0.03]
	**A → I → B**	**β = 0.09**	**0.01**	**0.018**	**2.37**	**[0.01, 0.06]**
Moderation	**A × PBC – SE**	**β = −0.20**	**0.07**	**0.003**	**−3.00**	**[−0.34, −0.07]**
	A × PBC – C	β = 0.06	0.04	0.154	1.43	[−0.02, 0.15]
	SN × PBC – SE	β = −0.04	0.06	0.463	−0.73	[−0.15, 0.07]
	SN × PBC – C	β = 0.07	0.04	0.117	1.57	[−0.02, 0.14]
	I × PBC – SE	β = −0.10	0.02	0.137	−1.49	[−0.06, −0.01]
	I × PBC – C	β = 0.00	0.02	0.997	0.00	[−0.04. 0.04]

*N = 196; SN = Subjective Norm; B = Behavior, PBC – SE = Perceived Behavioral Control – Self-Efficacy, PBC – C = Perceived Behavioral Control – Controllability, A = Attitude, I = Intention. Significant effects are highlighted in bold.*

The subsequent analysis of potential moderating effects of self-efficacy and controllability revealed that controllability did not significantly influence any of the investigated relationships, while self-efficacy had a significant impact on the relationship between attitude and intention. No moderating effects of self-efficacy were found on the relationships between subjective norm and intention or between intention and behavior.

### 5.4 Simple slope analyses

Finally, simple slope analyses were conducted to further examine the moderating effect of self-efficacy. To this end, the effects of the moderating variable were examined for different values of this variable (± standard deviation of 1.5; Hayes, 2013). The simple slope analysis revealed that the relationship between attitude and intention remained significant only for the levels low and medium (Table 4).

**Table 4:** Simple slopes of the moderation analysis for the relation between attitude and intention.

Moderation	Level	Standardized regression coefficient	Standard error	*p*	*z*	95%-CI [LB, UB]
Self-efficacy (A × PBC – SE)	low (−1.5 *SD*)	β = 0.27	0.06	< 0.001	4.89	[0.17, 0.41]
	medium (*M*)	β = 0.17	0.03	< 0.001	6.71	[0.13, 0.23]
	high (+1.5 *SD*)	β = 0.07	0.04	0.073	1.80	[−0.01, 0.14]

*N = 196; A = Attitude, PBC - SE = Perceived Behavorial Control - Self-Efficacy.*

## 6 Discussion

The aim of the current study was to theoretically ground and empirically examine the conditions for implementing gamification within the framework of the TPB. It was assumed that teachers’ attitude, perceived subjective norm, perceived behavioral control, and intentions play a decisive role in the application of this teaching method. The relationships between the investigated variables were examined using a path model with factor scores, which demonstrated a good model fit. The relationships examined in this model are discussed separately in the following sections.

### 6.1 Effects of attitude toward gamification on intention and behavior

The attitude toward the use of gamification in class was positive among the teachers examined in the current study (see Huizenga et al., 2017; Martí-Parreño et al., 2016; Pozo et al., 2022). They believe that gamification is useful, beneficial, and meaningful for the students’ learning processes. Moreover, teachers with a more positive attitude toward gamification were more willing to use this teaching method in their lessons (H1a). This effect of attitude on intention is in line with previous studies in educational contexts (Sánchez-Mena et al., 2016, 2017; Sajinčič et al., 2022) as well as with the assumptions of the TPB (Ajzen, 1991). Moreover, a moderating effect of self-efficacy, but not of controllability, which were used to measure perceived behavioral control, was observed (H4a). Hagger et al. (2022) did not find moderating effects of perceived behavioral control in the context of health behavior with regard to the relationship between attitude and behavioral intention (see Ajzen, 2020). Therefore, our results regarding controllability align with those of Hagger et al. (2022), whereas our results on self-efficacy are not in line with the results of their study. These inconsistencies between the current study and the study by Hagger et al. (2022) might be attributed to the fact that the studies were conducted in different contexts. As Hagger et al. (2022) point out, few studies have examined the moderating effect of perceived behavioral control on the relationship between attitude and intention, and those that have yielded inconsistent results. It should be noted that in most cases, previous studies have examined the direct effects of perceived behavioral control on intention (Hagger et al., 2022). At the same time, it must be borne in mind that perceived behavioral control was measured differently in the current study – via self-efficacy and controllability – compared to Hagger et al. (2022). Finally, regarding controllability, its low reliability may be another possible reason for the non-significant moderation, which we discuss later in this chapter.

Regarding the moderating effect of self-efficacy, our simple slope analysis revealed differences in the relationship between attitude and intention when comparing different levels of self-efficacy. The strongest relationship between the two variables occurs when self-efficacy is low. That is, a positive attitude toward gamification is of particular importance for the intention to use gamification if teachers feel little effective in its implementation. The positive attitude seems to result in teachers forming intentions even though they rate their skills as low. However, it could be that this intention does not translate into behavior due to low perceived self-efficacy. The correlation between attitude and intention is somewhat weaker, but still significant for a medium level of self-efficacy. Even with moderately perceived self-efficacy, a positive attitude still appears to be important for behavioral intention. When teachers are highly self-efficacious in the implementation of gamification, a positive attitude no longer seems to be important for the formation of intentions. As the current state of research is heterogeneous (see Hagger et al., 2022), these interesting correlations should be investigated in more detail in further studies.

Besides its effects on intention, attitude also had direct and indirect effects (via intention) on the teachers’ actual implementation of gamification in the classroom (H3a). These findings are in line with the assumptions of the TPB (see Ajzen, 2020). However, previous studies in educational contexts suggest a discrepancy between attitude and behavior (e.g., Basten, 2013; Gülsün et al., 2023). In Gülsün et al.’s (2023) study, this may be due to the incompatibility between the operationalization of attitude and behavior. Specifically, the assessment of attitude focused on inclusive education and the teaching of children with special educational needs, whereas behavior was assessed in relation to teaching students appropriate behaviors in various situations (Gülsün et al., 2023). According to Rossmann (2021), the relationship between attitude and behavior is stronger when both constructs share the same level of specificity and are therefore compatible. In the present study, the reference points of the items used to assess these variables were chosen to align—that is, the items asked about applying gamification in class.

In the context of organ donation, Basten (2013) identified such a gap when asking students about their possession of an organ donor card. She found that despite a positive attitude toward organ donation, only a few students actually had an organ donor card. Teaching units on the topic of organ donation were able to counteract this attitude-behavior gap (Basten, 2013). Tarfaoui and Zkim (2017) suggest that an attitude-behavior gap can be caused by social desirability, which reflects how strongly a person tends to present their attitude and behavior in a socially acceptable way. It is possible that the teachers in our study had heard about the positive effects of gamification and therefore thought that, as good teachers, they should adopt a positive stance, engage with the concept, and use this new approach in their lessons. The teachers’ perceived subjective norm could provide evidence for such assumptions (see Ajzen, 1991). This variable is discussed in the following section.

### 6.2 Effects of subjective norm on intention and behavior

The teachers’ perceived subjective norm was only slightly pronounced in the current study, suggesting that they tend to act somewhat independently of the opinion of their colleagues or other members of their school environment. This result is consistent with the results of Bourgonjon et al. (2013), which also dealt with gamification. However, it should be kept in mind that subjective norm is highly context-dependent and may vary between different school settings, which could explain discrepancies between studies.

Regarding its predictive effects, the current study revealed a positive effect of subjective norm on intention (H1b). That is, the subjective norm that teachers perceive seems to determine their intention to use gamification in their classes. This relationship was neither moderated by teachers’ self-efficacy regarding the implementation of gamification, nor by their perceived controllability in implementing this approach (H4b). The predictive role of subjective norm on intention is in line with the TPB and previous empirical evidence (Bourgonjon et al., 2013; Weber and Fiebelkorn, 2019). Bourgonjon et al. (2013) found a strong positive correlation between teachers’ perceived subjective norm and their intention to use gamification in future teaching. In our study, subjective norm was the weakest predictor. Similar weak effects were also found in a study by Weber and Fiebelkorn (2019), which examined the effects of preservice teachers’ perceived subjective norm on their intention to eat sustainably. Once again, it has to be borne in mind that this study did not focus on the implementation of measures in the school context, where external conditions may differ significantly from those in individuals’ private lives. In private settings, individuals might have greater control over their actions and can behave more consistently with their personal norms. Such differences can have an effect on the relationships assumed in the TPB (see Sutton, 1998).

If the focus of previous studies is broadened, findings on the predictive effect of the subjective norm on intention appear to be less consistent than one might expect. In Lübke et al.’s (2016) study, the subjective norm regarding the inclusion of children with special educational needs did not significantly predict the intention to use differentiated teaching strategies. Their study focused specifically on the perceived subjective norm in relation to the school management, which suggests that other members of the school community, such as colleagues, might have a more significant impact on teachers’ intention and behavior (Lübke et al., 2016). Credence for such assumptions might be found in the current study, which focused on teachers’ perceived subjective norm regarding their colleagues. The observed negative effect of subjective norm on the intention to use technology in the classroom in the study reported by Teo et al. (2016) contradicts our results. This discrepancy could result from differences in the teaching approaches examined (use of gamification vs. use of technology). In addition, societal changes regarding the use of technology since the publication of Teo et al. (2016) may have led to greater support for and broader application of technology in education. The use of technology in education has been particularly influenced by the COVID-19 pandemic (UNESCO, 2023). As a result, the perceived subjective norm regarding the integration of new technologies in the classroom may have shifted. To further investigate this, the study by Teo et al. (2016) should be replicated.

Besides predicting intention, we found no indirect effect of subjective norm on behavior via intention (H3b). First, this finding could indicate that other variables play a more significant role in the use of gamification in class (see Teo et al., 2016). This possibility is supported by the significant effects that we found for attitude and self-efficacy. However, the intention to use gamification was still positively predicted by the teachers’ perceived subjective norm. Second, as cited above, the social reference points considered when assessing their perceived subjective norm might be relevant to its effects on behavior. Our results might have differed if we had asked about the expectations of the school principal, students, or parents (see Lübke et al., 2016; Tran et al., 2023). Third, background factors, such as age or professional experience, may have influenced our results (Gülsün et al., 2023; Wang and Tsai, 2022). For instance, we found that as professional experience increases, the teachers’ intention to use gamified approaches in class tend to decrease (Table 2). Experienced teachers are often considered more adept and effective, which may explain why they might have a lower intention to apply new methods, preferring instead to rely on proven ones or habitual practices (see Hähn and Ratermann-Busse, 2020; Triandis, 1980). The correlations that we found lend credence to this assumption (see Table 2). However, we only found a correlation between intention and professional experience, but not between behavior and professional experience. This suggests that teachers, regardless of their level of experience, do seem to apply gamification in their teaching, at least to some extent.

In addition, we did not find moderating effects of perceived controllability or self-efficacy on the relationship between subjective norm and intention (H4b), which confirms the assumptions of Hagger et al. (2022). A key distinction in our study is that, unlike Hagger et al. (2022), we measured perceived behavioral control separately through controllability and self-efficacy. Ajzen (2002) differentiates between controllability as an environment-related variable, which reflects the influence of external factors on behavior, and perceived self-efficacy as a person-related variable, which represents an individual’s internal assessment of his/her own ability to perform a certain behavior (Ajzen, 2002). Both variables do not appear to affect the relationship between subjective norm and intention. However, more research is needed on these moderating effects due to a lack of research and mostly inconsistent findings (Hagger et al., 2022).

Besides potential moderating effects, perceived behavioral control can also have a direct effect both on intention and on the relationship between the intention to use gamification and its actual implementation in the classroom. These relationships are discussed in the following section.

### 6.3 Effects of perceived behavioral control on intention and behavior

To investigate perceived behavioral control, we assessed teachers’ self-efficacy as a person-related variable and controllability as an environment-related variable. Since studies using the TPB differ in how they measure this construct, comparisons with previous studies are only partially possible—a challenge that has already emerged in previous discussions. The participating teachers in our study generally reported high levels of both self-efficacy and controllability. That is, they feel capable of implementing gamification in their classes and believe they have the necessary skills. Moreover, most teachers believe that the decision to use gamification is largely within their control and that they can apply it independently of their environment (see Ajzen, 2002). However, it should be noted that their self-efficacy is less strongly pronounced than their perception of controllability. Therefore, supporting teachers’ self-efficacy may be an important starting point for fostering the use of gamification in the classroom. This assumption is supported by our finding that self-efficacy significantly predicts the intention to apply gamification (H1c). This result is consistent with the results of previous studies that investigated the relationship between perceived self-efficacy and teacher behavior for other types of instruction in classrooms (Klassen and Tze, 2014; Gülsün et al., 2023) as well as in line with Ajzen’s (2002) discussion. For example, Gülsün et al. (2023) showed that teachers’ self-efficacy regarding classroom behavior management positively and significantly predicted their ability to teach appropriate behaviors to students.

In contrast, controllability, as an environment-related variable could not be confirmed as a predictor of intention (H1c). It should be noted that we found only weak reliability for the items assessing controllability. A measurement instrument can only yield valid results if it demonstrates sufficiently high reliability (Döring and Bortz, 2016). Therefore, both the reliability and validity of this measurement can be limited. To assess controllability, we included one inverse item. Research suggests that inverse items can impair the assessment of variables (İlhan et al., 2024); for example, respondents may overlook such items in extensive questionnaires. This, in turn, could introduce bias into response behavior (see Bühner, 2021). Moreover, the inverse item in our study showed a low factor loading, which may have contributed to the low reliability (see Großmann and Randler, 2025). However, we decided to retain this item for conceptual reasons—removing it would have meant losing an important facet of the definition. Future studies could consider replacing this inverse item with a non-inverted one. In addition to measurement-related concerns, another possible explanation for our findings is that the participating teachers may have been reluctant to fully acknowledge that their environment limits their autonomy in lesson planning. This reluctance may have contributed to the absence of expected correlations between controllability and intention. How intention directly affects behavior is discussed in the following section.

### 6.4 Effects of intention on behavior

Our findings suggest that teachers’ intentions to use gamification do, in fact, translate into actual behavior (H2), which is in line with the assumptions of the TPB (Ajzen, 2020). However, previous studies have often focused solely on intention without examining behavior. Even when both variables were examined, many studies failed to find a significant connection (see Conner and Norman, 2022; Lübke et al., 2016; Urton et al., 2023). Sheeran (2002) describes this phenomenon as the *intention-behavior gap* and emphasizes that the relationship between these variables is complex rather than straightforward. The TPB assumes that the prediction of behavior depends not only on individual characteristics, but also on the contextual opportunities available for enacting the intended behavior (Ajzen, 1985; see also Sheeran, 2002). That is, if teachers lack sufficient control over the enactment of their intended behavior or are not provided with sufficient opportunities within the school environment, their intentions may not translate into action. This assumption is supported by Sutton (1998), who points out that an individual’s evaluation of situational factors plays an important role in predicting behavior and that intentions most likely vary depending on the context. To account for this, we examined whether perceived behavioral control moderates the relationship between intention and behavior, as suggested by Ajzen (2020). However, we did not find evidence for such moderating effects of controllability, which is not consistent with the assumption that context-specific factors, such as environmental control, influence this relationship (H4c). Sheeran (2002) points out that some behaviors are driven more by automaticity (Bargh, 1997) or habits (Triandis, 1980) than deliberate intentions. This might be particularly relevant when it comes to adopting and/or applying new teaching methods, especially when considering teachers’ workload and the limited time available to teach all curriculum-relevant content (see Zerndt, 2024). Due to these time and resource constraints, teachers may be more likely to rely on familiar teaching strategies that they can implement efficiently and habitually, rather than experimenting with new approaches.

Moreover, we did not find moderating effects of self-efficacy in the relationship between intention and behavior (H4c). This finding is not in line with previous studies, which have demonstrated that perceived self-efficacy is important for overcoming challenges in the teaching context (see Klassen and Tze, 2014). Self-efficacy might strengthen the link between intention and behavior by enabling teachers to translate their intentions into actions. Hagger et al. (2022) found such moderating effects of perceived behavioral control in the relationship between intentions and behavior (see Ajzen, 2020). However, it should again be noted that our findings may deviate due to differences in both the investigated context and the way perceived behavioral control was assessed. To our knowledge, no previous studies in the educational context have investigated the moderating effects of perceived behavioral control with this level of differentiation. We believe that this differentiated assessment of personal and environmental factors provides a more comprehensive insight into perceived behavioral control and should therefore be pursued in future studies.

Regarding the assessment of intentions and behavior, it is essential to consider that intentions can only predict behavior when both variables are measured with equal specificity (Fishbein and Ajzen, 1975). Given that gamification is a broad construct encompassing a range of approaches (see Cheng, 2020; Sailer et al., 2016), our assessment may not have been sufficiently specific. However, both constructs were rated with regard to gamification and were thus measured at the same level of specificity. Besides specificity, it has to be kept in mind that we applied self-reports to assess the intentions to enact gamification, which could limit predictive validity and weaken the intention-behavior relationship. Gollwitzer (2014) proposes examining implementation intentions since they are more likely to predict actual behavior. These intentions are assumed to be the most validated and frequently investigated method to close the intention-behavior gap and are assessed in reference to a specific situation in which the intended behavior will be enacted (Gollwitzer, 2014; Gollwitzer and Sheeran, 2006; Sheeran and Webb, 2016). Even though our study did not identify an intention-behavior gap, future studies could explore this alternative approach to measuring intentions.

### 6.5 Limitations and implications

Despite our important findings, there are limitations to our study. Firstly, the completion of the questionnaire was voluntary. This self-selection may have influenced the results, as the participating teachers may have a higher interest in the topic and thus have more positive attitudes and a greater intention to use gamification than those who chose not to complete the questionnaire. This assumption is also reflected in the large percentage of teachers in our study who actually use gamification (85%). This limitation may affect the generalizability of the results and the representativeness of the sample (Döring and Bortz, 2016). To allow for greater generalizability and representativeness, future studies should encourage teachers to participate in the study regardless of their interest in the topic or their (non-)implementation of gamification. Besides generalizability, the cross-sectional nature of our data does not allow us to draw conclusions about causal effects (Döring and Bortz, 2016). Therefore, future studies on the investigated correlations should be designed longitudinally.

Secondly, the validity of the applied test instrument needs to be discussed. Since we used items from two different sources for assessing our constructs (Francis et al., 2004; Jerusalem et al., 2009), validity might be limited. However, we believe that validity can be assumed for our test instrument for the following reasons: The items were taken from a widely used manual for assessing the constructs anchored in TPB (Francis et al., 2004) and a common scale book for assessing self-efficacy (Jerusalem et al., 2009). The combination of items from these two sources was necessary because, in our opinion, the manual for assessing TPB constructs does not cover all facets of self-efficacy. For this reason, the items in the manual (Francis et al., 2004) were supplemented with items from established scales for measuring self-efficacy (Jerusalem et al., 2009). In addition to the credibility of the used sources, the findings of our and previous studies suggest that the scales measure the constructs validly (see Aptyka and Großschedl, 2022; Lee et al., 2010; Luszczynska et al., 2005; Rothland, 2013; Scholz et al., 2002). Theoretically assumed correlations were found in our study as well as the cited studies (see Aptyka and Großschedl, 2022; Lee et al., 2010; Luszczynska et al., 2005; Rothland, 2013; Scholz et al., 2002; criterion validity). In addition to criterion validity, a further facet of validity can be demonstrated with our findings (factorial validity; Hartig et al., 2020). The confirmatory factor analysis shows that the items can be assigned to the corresponding latent variables and confirms the theoretical structure (see Ajzen, 1991; Graf, 2007). We therefore assume that the constructs were assessed with sufficient validity for the analysis in our study. Merely the variable controllability requires further investigation and should be viewed with caution. This variable has already been discussed and suggestions for adjustments in future studies have been given.

Thirdly, it should be noted that the data collected are self-reports by the investigated teachers. Self-reported data can be socially biased; that is, teachers might have rated the items socially desirable (van de Mortel, 2008). If teachers perceive that the people in their social environment think positively about gamification and may feel pressured to use such new methods in their teaching, it could be that they have rated the investigated constructs more positively than they actually feel about them and might have stated that they use gamification even though they do not. The constructs we assessed could evoke such feelings. However, it was made clear in our survey that we could not draw any conclusions about schools or individuals and that the data was completely anonymous. This procedure should have enabled a free and honest rating of the scales. Nevertheless, we cannot rule out a social bias in our data. To verify the plausibility of the statements of the surveyed teachers and to provide a comprehensive view of the reality of teaching, student feedback could be obtained in future studies (see Gärtner, 2013). Differences could be assumed here, as, for instance, Göllner et al. (2016) show that there is a low agreement between teachers’ self-reports and other data sources. Classroom observations with external observers could be implemented as an alternative (Praetorius, 2013; Praetorius et al., 2017). However, this alternative method can affect the observed teacher and students in class and lead to biased data as well (Praetorius, 2013; Praetorius et al., 2017).

Fourthly, future studies should consider background factors that indirectly influence intention via attitude, perceived subjective norm, and perceived behavioral control (Graf, 2007; Rossmann, 2021). For instance, a subject-specific study would be possible, as different subjects and related topics inherently offer different conditions for the implementation of gamification. In scientific subjects, simulation games can, for example, be used to illustrate complex scientific topics, which are usually unavailable in other subjects (Adeyele, 2024; Hilton and Honey, 2011). Experience with gamified learning environments and the experience with games during leisure time can be cited as further influencing factors (see Rossmann, 2021). Such experiences can impact teachers’ attitude and self-efficacy, as well as their intention and behavior in class and should therefore be considered in future studies (Pozo et al., 2022).

Regarding the demographic variables that were already investigated in the current study, some interesting correlations were revealed. We found significant correlations between the type of school and attitude, controllability as well as subjective norm (Table 2). The negative correlation between type of school and attitude indicates that teachers in schools with lower tracks express a less positive attitude toward the use of gamification than teachers in schools with higher tracks. However, the negative correlation between type of school and controllability at least suggests that teachers at schools with lower tracks have a certain freedom in terms of their teaching (see Ministry for School and Further Education of the State of North Rhine-Westphalia, 2012). The negative correlation with attitude might be attributed to the fact that implementing gamification, for instance, in elementary schools, is associated with several challenges, including a lack of resources and the large amount of time required to integrate this method (Sáez-López et al., 2023). It might also be that teachers from different types of schools have different attitudes toward gamification due to the availability of materials. Nevertheless, it must be borne in mind that the negative correlation found is rather weak and that diverse schools were investigated, which are not represented in equal proportions in the sample analyzed. We will discuss this in more detail in the next section. Regarding subjective norm, we found a higher agreement for teachers at lower-track types of school than teachers at higher-track types. It might be that due to the special challenges at lower-track school types, such as more intensive social integration and instructional support, teachers at these schools perceive higher expectations and social pressure to integrate new concepts (see Robert Bosch Stiftung, 2023; Sutter-Brandenberger et al., 2019). Gamification may be one of these new concepts to foster social integration and provide additional instructional support.

With regard to the type of school, it has to be kept in mind that a large proportion of the teachers surveyed teach at a higher-track school. This was not a deliberate choice but may have led to a bias in our data and limits the generalizability of our findings. Future studies could take this limitation into account and, when recruiting the sample, ensure that there is a balance of school types and other characteristics of the sample. For instance, it could be pointed out in the letter to potential participants that the survey is aimed at teachers from different types of schools who do or do not (yet) implement gamification. A more balanced sample would allow more meaningful conclusions to be drawn concerning school type. In a sufficiently large sample, the tested models in our study could be calculated separately for each type of school and compared with each other. School-type-specific differences in conditions for and challenges in the implementation of gamification might be presumed based on the correlations we found.

Our results further show negative correlations between gender and the variables attitude and self-efficacy (Table 2). Furthermore, we found a positive correlation between gender and behavior. With regard to the latter, it has to be kept in mind that Cramer’s V cannot take on negative values. The distribution of gender in the behavior categories “yes” and “no” revealed that female teachers implement gamification more often than male teachers. Moreover, they have a more positive attitude regarding gamified learning environments and state a higher level of self-efficacy than male teachers. Previous studies have shown inconsistent results regarding gender-specific differences in the attitude toward gamification or similar teaching approaches (see Huffman et al., 2013; Martí-Parreño et al., 2016). Jent and Janneck (2016) found that females perceive greater social benefits from the use of gamification (see also Koivisto and Hamari, 2014). It could be that such positive experiences in dealing with gamification lead to a more positive attitude toward gamification. The attitude toward gamified learning environments is positively related to perceived self-efficacy (see Erdem, 2015; Yau and Leung, 2018). Both variables, in turn, positively affect intention and, ultimately, behavior (see Ajzen, 1991, 2002). Due to these correlations between the variables, it is not surprising that the correlation with gender is evident in all of these variables, except for intention. These correlation patterns can provide indications for the validity of our study. When discussing gender effects in our study, it should, however, be noted that we have a large proportion of female teachers in our sample.

Finally, our findings do not allow any conclusions to be drawn about specific gamification approaches. Following the question regarding their behavior (“I use gamification in my teaching.”), we allowed the teachers to justify their previous selection. The approaches that they listed ranged from video games to smaller playful elements, such as quizzes. These approaches vary in complexity and effort. Future studies could refer to specific gamification approaches to be able to make statements about the implementation of certain approaches in class.

## 7 Conclusion

The current study aimed to gain a comprehensive insight into the conditions of the use of gamification approaches in class. With this study, we theoretically support as well as expand previous empirical findings and were able to identify significant teacher variables for the use of gamification in class. The variables of the TPB were confirmed as predictors of the intention to use gamification in class, except for controllability. In addition, we found effects of the teachers’ intentions to use gamification on their actual behavior in class. Some of the assumed mediating (intention) and moderating effects (self-efficacy) were confirmed. Contradictory findings with previous studies may have resulted from the fact that these studies applied the TPB in other contexts under different conditions or used other instruments and reference points to measure the variables. This is especially true for subjective norm and perceived behavioral control.

As positive effects of gamified learning environments are frequently cited (Bourgonjon et al., 2013; O’Brien and Pitera, 2019), the intention of teachers to use such approaches in their classes should be encouraged. Our findings indicate that teachers’ attitude, their perceived subjective norm as well as their perceived self-efficacy could be focused on in interventions to foster the intention to use gamification. Our findings show that such intentions can translate into actual behavior.

Van Twillert et al. (2020) point out that helping educators gain both knowledge and experience with gamification in further education and training can positively influence the actual incorporation of gamification into learning environments. The provision of relevant information about gamification can encourage teachers to rethink their personal attitudes toward gamified learning environments, especially when the reasons for neglecting them stem from a lack of information (Becker, 2007). Furthermore, providing knowledge and experience can influence perceived behavioral control by helping teachers overcome barriers related to a lack of knowledge and experience (Ajzen, 1991). The application of gamification in class can also be positively affected by providing sufficient external resources (van Twillert et al., 2020; Wang and Tsai, 2022). These resources can enhance teachers’ self-efficacy and self-confidence in using gamification approaches (van Twillert et al., 2020; Wang and Tsai, 2022). Teacher training that includes materials or tools that support the development of gamified units can help teachers successfully implement gamification in the classroom (van Twillert et al., 2020). In our study, 87.8% of the participating teachers stated that they would participate in such training opportunities. Finally, if the use of gamification is endorsed and applied by significant others, such as colleagues, intentions and behavior can be positively influenced (Ajzen, 2020). That is, a reference group with positive attitudes toward gamification is needed to share positive experiences, attitudes, and success stories with other educators (van Twillert et al., 2020). However, training can have a limited influence on the perception of subjective norm. Moreover, this perception may vary depending on the grade level considered (Teo et al., 2016). Therefore, future studies might investigate gamification with regard to different grade levels and reference groups to give a more differentiated picture of the conditions for implementing gamification in class, ultimately helping to maximize the pedagogical potential of gamified learning environments.

## Data Availability

The raw data supporting the conclusions of this article will be made available by the authors, without undue reservation.
